# Rapid, Tailored Dietary and Health Education Through A Social Media Chatbot Microintervention: Development and Usability Study With Practical Recommendations

**DOI:** 10.2196/52032

**Published:** 2024-12-09

**Authors:** Shahmir H Ali, Fardin Rahman, Aakanksha Kuwar, Twesha Khanna, Anika Nayak, Priyanshi Sharma, Sarika Dasraj, Sian Auer, Rejowana Rouf, Tanvi Patel, Biswadeep Dhar

**Affiliations:** 1 Saw Swee Hock School of Public Health National University of Singapore Singapore Singapore; 2 School of Global Public Health New York University New York, NY United States; 3 College of Computing, Data Science, and Society University of California, Berkeley Berkeley, CA United States; 4 University of Minnesota Medical School Minneapolis, MN United States; 5 Department of Family and Community Medicine University of Illinois College of Medicine Rockford Rockford, IL United States; 6 Department of Human Ecology University of Maryland Eastern Shore Princess Anne, MD United States

**Keywords:** social media, chatbot, conversational agent, intervention, diet, health education, feasibility, microintervention, innovation, dietary education, social media chatbot, public health professional, young adult, Asian, curriculum

## Abstract

**Background:**

There is an urgent need to innovate methods of health education, which can often be resource- and time-intensive. Microinterventions have shown promise as a platform for rapid, tailored resource dissemination yet have been underexplored as a method of standardized health or dietary education; social media chatbots display unique potential as a modality for accessible, efficient, and affordable educational microinterventions.

**Objective:**

This study aims to provide public health professionals with practical recommendations on the use of social media chatbots for health education by (1) documenting the development of a novel social media chatbot intervention aimed at improving dietary attitudes and self-efficacy among South Asian American young adults and (2) describing the applied experiences of implementing the chatbot, along with user experience and engagement data.

**Methods:**

In 2023, the “Roti” chatbot was developed on Facebook and Instagram to administer a 4-lesson tailored dietary health curriculum, informed by formative research and the Theory of Planned Behavior, to 18- to 29-year-old South Asian American participants (recruited through social media from across the United States). Each lesson (10-15 minutes) consisted of 40-50 prescripted interactive texts with the chatbot (including multiple-choice and open-response questions). A preintervention survey determined which lesson(s) were suggested to participants based on their unique needs, followed by a postintervention survey informed by the Theory of Planned Behavior to assess changes in attitudes, self-efficacy, and user experiences (User Experience Questionnaire). This study uses a cross-sectional design to examine postintervention user experiences, engagement, challenges encountered, and solutions developed during the chatbot implementation.

**Results:**

Data from 168 participants of the intervention (n=92, 54.8% Facebook; n=76, 45.2% Instagram) were analyzed (mean age 24.5, SD 3.1 years; n=129, 76.8% female). Participants completed an average of 2.6 lessons (13.9 minutes per lesson) and answered an average of 75% of questions asked by the chatbot. Most reported a positive chatbot experience (User Experience Questionnaire: 1.34; 81/116, 69.8% positive), with pragmatic quality (ease of use) being higher than hedonic quality (how interesting it felt; 88/116, 75.9% vs 64/116, 55.2% positive evaluation); younger participants reported greater hedonic quality (*P*=.04). On a scale out of 10 (highest agreement), participants reported that the chatbot was relevant (8.53), that they learned something new (8.24), and that the chatbot was helpful (8.28). Qualitative data revealed an appreciation for the cheerful, interactive messaging of the chatbot and outlined areas of improvement for the length, timing, and scope of text content. Quick replies, checkpoints, online forums, and self-administered troubleshooting were some solutions developed to meet the challenges experienced.

**Conclusions:**

The implementation of a standardized, tailored health education curriculum through an interactive social media chatbot displayed strong feasibility. Lessons learned from challenges encountered and user input provide a tangible roadmap for future exploration of such chatbots for accessible, engaging health interventions.

## Introduction

### Health Education Interventions: an Emerging Need for Innovation

Health education interventions are crucial for reducing disease burdens and promoting overall health by equipping individuals with the knowledge and skills to address socioecological health determinants, such as attitudes, behaviors, and the environment [1]. Common methods include interactive instruction (eg, workshops) and passive instruction (eg, pamphlets) [1-3]. For example, nutrition education delivered through emails and in-person workshops has been associated with improved nutritional knowledge, healthier dietary habits, and better health outcomes [4].

South Asian individuals in the United States face a disproportionate burden of diet-related diseases, such as type 2 diabetes and heart disease, affecting even younger adults compared to other racial and ethnic groups [5-8]. There is a particularly pressing need for tailored nutrition interventions for communities such as South Asian American individuals, given that existing educational interventions may not reflect the types of foods consumed (including methods of food preparation) within the community [9]. However, many traditional health education methods are resource-intensive, requiring in-person visits and long-term commitment, which can be costly and inaccessible, particularly for underserved populations [10,11].

A promising alternative is the “micro-intervention” approach, which involves brief (10-20 minutes) and adaptable exercises that quickly provide targeted solutions [12,13]. Studies have shown that microinterventions can improve short-term dietary behaviors, such as increasing fruit and vegetable intake and reducing fat consumption [14,15]. These interventions offer interactive feedback to maintain engagement, presenting an opportunity for developing affordable, accessible, and impactful health education methods tailored to specific communities.

### Using Social Media in Health Interventions: An Accessible, Affordable, and Efficient Tool

Social media has emerged as a powerful tool for health promotion, offering cost-effective, timely delivery of messages. With 4.26 billion global users, platforms like Facebook (2.9 billion users), WhatsApp (2 billion users), and Instagram (2 billion users; Meta) are particularly popular [16-18]. In the United States, there are 100 million social media users, including 84% of young adults (aged 18-29 years) and 81% of adults (aged 30-49 years) [18,19]. Asian American individuals, including South Asian American individuals, report high social media use, with studies highlighting its popularity for accessing health and diet information [20-22].

Social media–based health interventions can reduce the costs of in-person interactions and provide services anytime [13]. These interventions often use passive educational methods, such as sharing health-related resources, reminders, and infographics. More active methods, like 2-way SMS text message or video chat and discussions on social networking sites, promote interactions between participants, peers, and health workers [23]. Social media has also shown promise in promoting healthy dietary behaviors; a systematic review found that social media–based dietary interventions significantly reduced dietary fat consumption [24,25]. However, most research has focused on White populations or high-income countries, with limited studies on social media–based health education in low- and middle-income countries or among racial and ethnic minority groups in high-income countries, such as South Asian American individuals [9,25,26].

### Social Media Chat Bots: A New Frontier for Health Education

Chatbots—nonhuman conversational agents—are increasingly used in various sectors, including customer service, education, and health care, due to their efficiency in providing information and maintaining engagement. In health care, chatbots assist with diagnosis, treatment, monitoring, support, and health promotion [27]. There are two main types: rule-based and unconstrained chatbots. Rule-based chatbots follow predetermined rules and are easy to implement, accounting for 56% of lifestyle chatbot interventions [10]. Unconstrained chatbots simulate human conversation, offering flexibility but requiring more human oversight to maintain quality.

While chatbot-based health interventions are growing, they often focus on limited issues, such as mental health and COVID-19 [28]. One diet-related study used a daily messaging intervention on Facebook Messenger (Meta) to reduce red and processed meat consumption, achieving significant attitude changes but lacking conversational elements [29]. A 2021 review of 62 health interventions using chatbots found that only 6 used social media platforms, and no chatbot microintervention on social media has used a standard curriculum to deliver tailored lessons on behavioral and attitudinal contributors to noncommunicable diseases, including diet [10]. Exploring the feasibility of social media chatbots to deliver standardized educational content could help translate validated curriculums on chronic disease prevention to broader audiences [30,31].

To meet this pressing need for innovation in health education strategies coupled with the growing exploration of social media chatbots in health research, this study describes the development and implementation of a social media chatbot (named “Roti”) aimed at rapidly providing education related to dietary attitudes and self-efficacy among South Asian American young adults. Moreover, in a 2022 scoping review of chatbots used in public health research, authors explicitly highlighted the need for more research describing the implementation and technical experience of using chatbots in public health programs [28]. By documenting the applied experiences of this chatbot intervention, this paper aims to provide a practical roadmap for public health professionals interested in leveraging social media chatbots as a tool for rapid health education.

## Methods

### Lesson Development

The primary objective of this health education intervention was to promote healthy dietary attitudes and self-efficacy among South Asian American young adults. To inform lesson development, formative research was conducted to characterize the diet of South Asian American young adults [32,33] and identify salient influences in their dietary behaviors [20]. Specifically, this formative research involved (1) quantitatively analyzing the healthfulness of the dietary behaviors of all Asian Indian young adults surveyed in the 2015 National Health Interview Survey (n=175), (2) qualitatively analyzing the socioecological drivers of South Asian eating behaviors through interviews with South Asian American young adults in 2021 (n=32), and (3) quantitatively analyzing the food consumption patterns of South Asian Americans through a 2020 nationwide survey (n=570). Key areas of concern related to dietary attitudes and self-efficacy emerged from these findings, which were then disentangled using constructs of the Theory of Planned Behavior (TPB). Although there are various behavior change theories, the TPB was chosen given that it has been shown to be instructive in dietary research [34,35], including among South Asian individuals [36], and has also been identified as an example of a behavior change model that can be integrated into chatbot-based health interventions [37]. Although concerns related to all 3 central constructs of the TPB were identified (attitudes, subjective norms, and perceived behavior control), given that such individual-oriented chatbot interventions are likely less suitable to influence the drivers behind subjective norms (ie, relationships with friends and family) compared to other interventional modalities (eg, group-based sessions), this intervention focused more on dietary attitudes and perceived behavioral control. In total, four key areas of concern were identified: (1) limited understanding of how specific dietary behaviors can shape short-term and long-term health outcomes; (2) limited motivation to prioritize healthy eating as a South Asian individual and as a young adult; (3) limited confidence in the ability to cook healthy, flavorful foods while minimizing time, effort, and cost; and (4) limited confidence in navigating grocery stores to make healthful, flavorful food purchases while minimizing time, effort, and cost.

Each of the identified concerns was developed into an independent lesson. First, a team of researchers led by behavioral health scientists (both with PhDs in public health and training in young adult, South Asian, and nutritional health) conducted an analysis of published resources relevant to the diet of South Asian American young adults, which was used to supplement the insights from the aforementioned formative research (the foundation of the lesson content). This search was conducted as a narrative review using snowball sampling to identify any relevant scientific literature, nutritional guidance, and online resources relevant to each concern (prioritizing literature published within the last 10 years). All this information was then synthesized into a script that aimed to interactively convey the information by emulating a texting conversation (tailored to the tenor of a South Asian young adult). Specifically, each lesson consisted of a set of 40-50 texts of 20-50 characters each, along with images and diagrams to illustrate the lesson content, as well as between 12-16 multiple-choice and open-response questions spaced across the texts to help the participant engage with the course content. Once the script was developed, it was intensively pilot-tested through Facebook and Instagram messaging among the researchers to determine the appropriate time-spacing between texts to ensure readability (ultimately 3-12 seconds, depending on the amount of information in each text or image). Responses to the questions asked during each lesson did not influence the sequence of the texts provided, as their objective was to encourage participants to engage with and critically reflect on the course content in the context of their own lives. Particular attention was also made to ensure nutritional guidance was nuanced and sensitive to issues of disordered eating, a parallel and equally pressing issue within the South Asian community [38]. All scripts were reviewed by a registered dietician with expertise in South Asian health.

### Recruitment

Participants were recruited by outreach and advertising on diverse online platforms, including social media (Facebook, Instagram, Twitter, etc.) and email listserves. Recruited participants were first provided a link to the screening questionnaire. The eligibility criteria included (1) being aged 18-29 years old at the time of the survey, (2) self-identifying as South Asian (including Indian, Pakistani, Bangladeshi, Sri Lankan, and Nepali), (3) currently living in the United States, (4) being proficient in English, and (5) having either a Facebook or Instagram account to participate in the study. The screening questionnaire was followed by obtaining participants’ informed online consent and a pretest survey. Following the pretest survey, if participants had not initiated a conversation with the chatbot on either Facebook or Instagram, at least 2 reminders were sent via email (at 3- and 5-day intervals, respectively). Participants who had initiated a conversation with the chatbot but had not completed the posttest survey were similarly sent 2 reminders by both Facebook or Instagram and email (at the aforementioned time intervals). Participants were considered dropouts if they either informed their desire to exit the study by Facebook, Instagram, or email or if they had blocked the chatbot. Participation in the study involved only conversing with the automated chatbot; research staff only communicated with participants via the chatbot account to resolve issues during participation or to send reminders to complete a new lesson or submit the posttest survey.

### Data Collection

A pretest and posttest survey was provided to participants, which was collected by Qualtrics and stored in a protected database accessible only to the research team (and disconnected from Facebook or Instagram). The pretest survey included questions on demographic characteristics, acculturation, dietary acculturation, dietary healthfulness (measured through the 8-item Starting The Conversation [STC] dietary assessment tool [39], ranging from 0=lowest to 16=highest for dietary healthfulness), dietary attitudes, and self-efficacy. Details on the diet-related survey items have been described in detail in a separate impact evaluation of this intervention [40,41] (B Dhar, unpublished data, 2024). The posttest survey included the same questions on dietary attitudes and self-efficacy, along with questions to measure the usability and overall experience of the chatbot. Usability was measured through the 8-item User Experience Questionnaire (UEQ) [42]. The UEQ, which has been used in past evaluations of health technology interventions, assesses the user experience of a novel technology through its pragmatic quality (how supportive, easy, efficient, and straightforward it is) and hedonic quality (how exciting, interesting, inventive, and leading edge it is) from a scale from –3 to 3. The average score can be used to identify an overall positive evaluation (0.8 or above), neutral evaluation (0.8 to –0.8), or negative evaluation (–0.8 or below). Participants were also asked how strongly they agreed (from 1 to 10) on the following statements based on their experience of the intervention: (1) I learned something new, (2) I learned something helpful, (3) the content was relevant to me, (4) I would use it again, (5) I would prefer this to other ways of getting nutrition education or resources, and (5) I would use this to get education or resources on other health topics. Finally, participants were provided 2 open-response questions on what their favorite part was about chatting with the chatbot and if there was anything they would like to see changed to improve their experience of the chatbot.

### Variables of Interest

Although the content of the Facebook and Instagram conversations with the chatbot themselves were not analyzed, other types of metadata were examined, including which lessons the participants completed with the chatbot, when they began each lesson, how many responses they provided to the chatbot questions, and qualitative observations of how they participated in each lesson and conversed more generally. These qualitative observations included whether the participant was engaged and provided consistent, meaningful responses during each lesson, whether they faced any glitches or issues during the lesson and what was done to address them, and whether they had to make use of any of the main menu futures (eg, to get help, restart lessons, view sources, etc). To ensure participants felt comfortable engaging meaningfully with the chatbot during lessons, aside from making notes consistently and purposefully irrelevant or meaningless responses, the actual content of answers participants provided to the chatbot during lessons was not systematically analyzed. Multiple steps were also taken to optimize the reliability of the data. First, to avoid bots, the screening questionnaire also included a bot-detection question. Second, although study eligibility was primarily determined through the self-reported responses in the pretest survey, the emails, names, and Facebook or Instagram profiles of participants were also collectively inspected to identify potentially fraudulent responses. Third, using published guidelines, the actual survey data and engagement with the chatbot were evaluated in tandem with the aforementioned information to identify potentially fraudulent or meaningless data.

### Data Analysis

Data from the pretest, posttest, and Facebook or Instagram conversation metadata were assessed as part of this study. While this study focuses on user experience and engagement data, a separate study is being conducted to examine pre- and posttest changes in TPB and diet-related outcomes from this study. Postintervention survey data and Facebook or Instagram conversation metadata were descriptively analyzed, including using chi-square tests to examine characteristics of positive and negative user experiences. Postintervention open response data on user experience was qualitatively analyzed by identifying emergent themes related to participants’ favorite aspects of the chatbot and what they felt should be involved. All themes were independently reviewed by at least one other researcher, and any discrepancies were addressed through group consensus. In addition, researcher observations were qualitatively analyzed by identifying emergent themes related to challenges encountered and solutions implemented from notes taken during team meetings or any notes taken by those involved in the chatbot development and implementation; all themes were similarly independently reviewed by at least one other researcher.

### Ethical Considerations

Informed, online consent was taken of all participants, which involved asking participants to read a consent form and click “I agree” before commencing the pretest survey. All identifiable data collected as part of the study were stored in a password-protected shared drive only accessible by members of the research team. Aside from participants’ Facebook or Instagram handles, the data from participants’ social media profiles were not recorded or analyzed. Upon completion of data collection, all participant data were anonymized using a study ID, and any identifiable information was securely deleted. To incentivize participation, participants were also entered into a raffle to receive a US $50 gift card. Importantly, although data from social media profiles or messages were not systematically analyzed, within the consent form, participants were informed that the research team would still be able to see this information during the study and would use this to verify study completion (including checking for signs of fraudulency). All data collection methods and study materials were approved by the University of Illinois College of Medicine Rockford Institutional Review Board (approval 2034863).

## Results

### Final Chatbot Configuration

**Figure 1:**
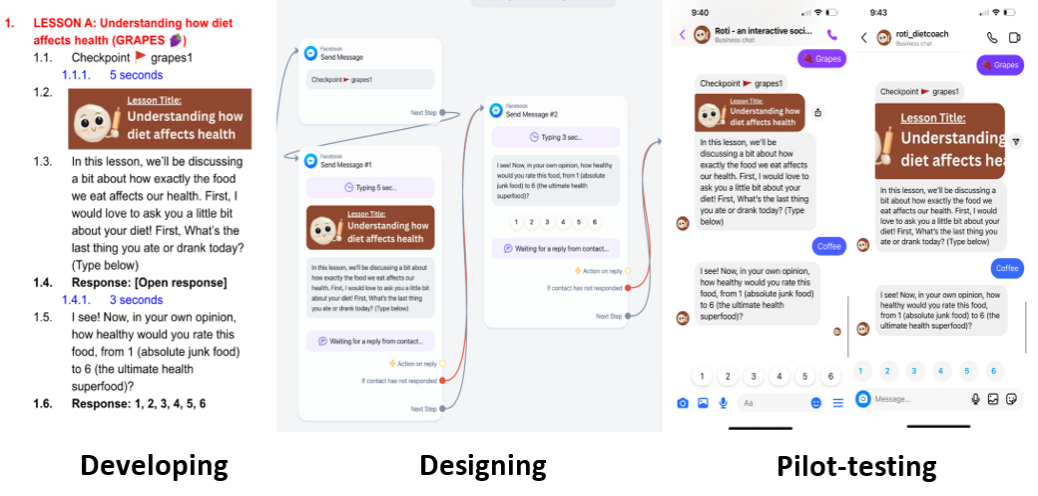
Process of developing and implementing lessons for the “Roti” social media chatbot intervention.

The final lesson script was integrated into an automated chatbot flow sequence using Manychat, a chatbot service integrated with various social media platforms, including Facebook and Instagram Messenger (Figure 1). Although there are many available chatbot software that could have been used for this study, Manychat was chosen given that (at the time of the study) it was among the more popular software, had been used in past health research [43,44], and had various available online resources and tutorials to facilitate its use. The chatbot, named “Roti,” was configured identically on both Facebook and Instagram Messenger, except that the Instagram version’s welcome message included a notice that it would only work on mobile devices. For open-response and multiple-choice questions, the chatbot waited 1-2 minutes for a response before moving to the next text rather than waiting indefinitely. This configuration was chosen because (1) lesson texts were short and interconnected, intended to be completed in one sitting (participants were informed); (2) varying participant engagement levels allowed the impact of engagement to be evaluated; and (3) there was a need to prevent lags and disruptions caused by waiting for user responses to every question (discussed in the *Results* section).

**Figure 2:**
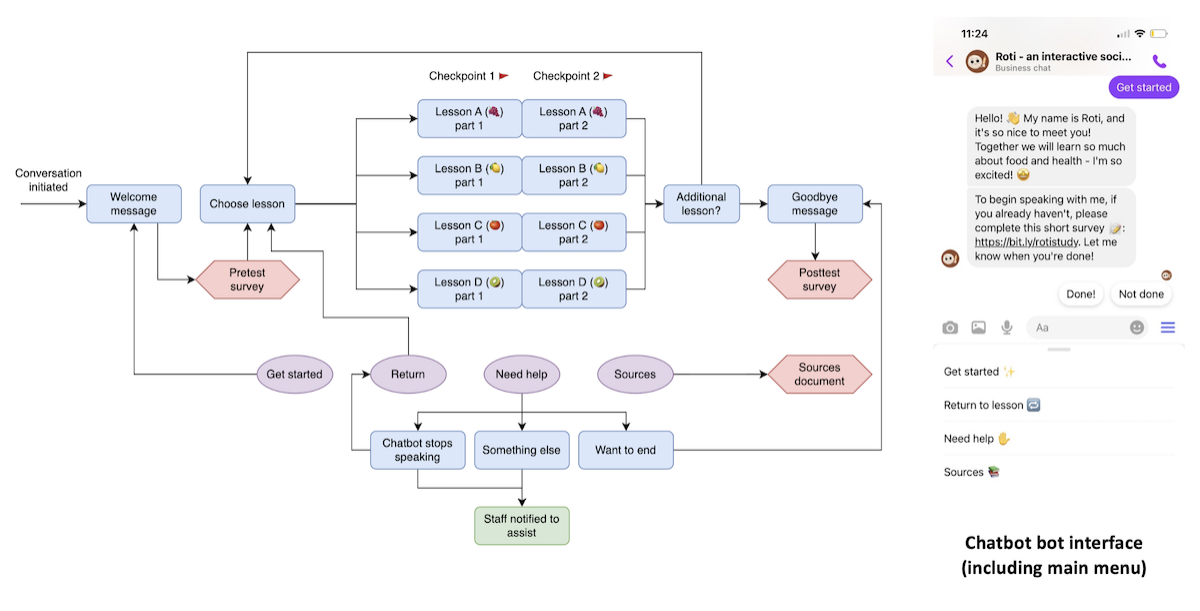
Participation flow sequence for the “Roti” social media chatbot intervention.

An overview of the participant experience of this chatbot intervention is visualized in Figure 2. Each lesson was given a codeword (a fruit) for ease of user experience. Before participation or upon initiating a conversation with the chatbot, participants were instructed to complete a preintervention survey, which included questions about dietary attitudes and self-efficacy corresponding to the content of each lesson. At the end of the survey, participants were provided 2 codewords identified as their greatest priority based on survey responses. Participants returned to the chatbot, entered their first codeword, and completed a lesson. In addition, 2 checkpoints were added at the start and in the middle of these lessons as a fail-safe in the case of the chatbot. Upon completing a lesson, participants were able to enter their second codeword to complete an additional lesson. Once participants completed 2 lessons or chose to conclude, they were presented with a goodbye message and a link to a postintervention survey. Importantly, the chatbot was also able to leverage Facebook or Instagram’s “main menu” feature to provide participants with a set of consistently available resources. This main menu included a “Get Started” button to return to the welcome message and a “Return” button to begin a new lesson of choice. In case of any issues or questions during the chatbot experience, participants were also able to select “need help,” which provided troubleshooting guidance. This guidance was informed by extensively pilot-testing the chatbot before implementation, identifying potential issues that could occur during the user experience, and developing solutions that could be self-implemented. If a participant was unable to resolve an issue or had any additional questions, then a member of the research team was able to intervene and directly communicate with the participant through the same account. Finally, participants could select “sources” to be provided a link to a document describing how the lesson content was developed and all relevant sources.

### Participants

Overall, 248 individuals completed the eligibility screener and were deemed eligible for the study, of which 231 (93.1%) completed the preintervention survey, and 176 (71%) commenced at least one lesson with the chatbot; 1 individual was excluded from this sample due to ineligibility (self-reported ethnicity was not from a South Asian or South Asian diasporic community), and 7 were excluded due to potential fraudulent self-reported South Asian status (information on participant’s social media profile did not reflect their self-reported South Asian ethnicity), leaving a final analytic sample of 168 (Multimedia Appendix 1). Out of this analytic sample, 116 (69%) also completed the postintervention survey. Sensitivity analyses (using chi-square and one-way ANOVA tests) revealed no significant differences in the sociodemographic characteristics of participants who did and did not complete at least one lesson with the chatbot and those who did and did not complete the postintervention survey. Participants had a mean age of 24.5 (SD 3.1) years; were majority female (129/168, 76.8%), born outside the United States (95/168, 56.5%), highly educated (89/168, 53% master’s degree or above), and Indian (119/168, 70.8%); and reported midlevel dietary healthfulness (STC: mean 6.94, SD 2.14).

### Engagement and User Experience

There was an approximately even split between participants who used Facebook (n=92, 54.8%) and Instagram (n=76, 45.2%) to participate in the intervention; compared with Facebook users, Instagram users, on average were 1.8 years younger (*P*<.001), had lower educational attainment (20/76, 26.3% vs 11/92, 11.9% some college, associate’s, or high school degree; *P*=.04), and had higher acculturation (4.0 vs 3.8 acculturation score; *P*=.04) (data not shown). Participants, on average, completed 2.6 of the lessons and spent an average of 13.9 minutes on each lesson (Multimedia Appendix 1). Engagement during lessons was also moderately high, with participants, on average, answering 75% of all questions asked by the chatbot.

Overall, participants reported a positive experience with the chatbot (UEQ 1.35; 81/116, 69.8% positive evaluation). However, its pragmatic quality (UEQ 1.59; 88/116, 75.9% positive evaluation) was notably higher than its hedonic quality (UEQ 1.11; 64/116, 55.2% positive evaluation). Participants had the strongest agreement that the chatbot was relevant to them (8.53) and that they learned something new (8.24) and helpful (8.28). Although most participants agreed to prefer this chatbot to other ways of receiving nutrition information (7.30), there was greater agreement on willingness to use the chatbot to receive information on other health topics (7.72). Overall, participant user experiences did not significantly differ across specific demographic categories or levels of engagement with the chatbot (Multimedia Appendix 2), except that those reporting positive hedonic quality were slightly younger (*P*=.04). However, UEQ scores consistently correlated with other indicators of the overall experience of the chatbot. Analyses of qualitative user experience data revealed that many participants appreciated the cheerful, positive, and supportive way in which the chatbot conversed with them and that the content was relevant, informative, engaging, and interactive (Table 1). A participant, for example, noted:

I loved how interactive it was, and how we were provided with evidence-based information and studies. I appreciated that we were sent links to videos as well.

**Table 1:** Summary of participant experiences of the “Roti” social media chatbot microintervention.

Question	Participant experiences
What was your favorite part?	Informative, relevant, and new content Cheerful, positive, and supportive tenor Sense of novelty Feeling of talking to a real, relatable person Engaging format and interactivity Rapid, easy, and efficient way to get information Use of visual media and graphics Including links for additional videos and resources Personalized questions during lessons
What would you like to see improved?	Adjusting the length, timing, and tenor of chatbot texts to improve readability Integrating with other social media, online, or text-based platforms Integrating AI^a^ to further personalize chatbot responses to user input Ability to pause and restart lessons, check lesson progress Expanding lesson content to include further personalized, substantive resource provision

^a^AI: artificial intelligence.

In terms of areas of improvement, some participants suggested adjusting the length, timing, and tenor of the chatbot texts, although the directionality of these recommendations sometimes differed between participants; for example, while many expressed a need for longer gaps between texts or more use of emojis, some also expressed wanting shorter gaps and less use of emojis. Some recommendations also centered around expanding and personalizing the lesson’s content, including integrating an artificial intelligence (AI) system to provide more tailored responses to user input. A participant, for example, noted:

I think making it more responsive to the actual answers and personalizing it [would be good]. I would love to be able to ask it questions as well.

### Challenges and Solutions

While developing and implementing the social media chatbot, some challenges were encountered, which were iteratively addressed by developing multiple different solutions (Table 2). Having a strong awareness of the features and functionalities of the third party chatbot software (Manychat) and Facebook/Instagram messenger was crucial in developing efficient workarounds to any limitations in these platforms, which may have disrupted user experience. While much of this information was available through online resources published by these platforms, online communities of social media chatbot users were very informative in developing potential troubleshooting strategies (eg, an active Facebook group of Manychat users was used to identify which issues may have been explained by server outages or platform glitches, and how different Manychat users addressed these sporadic complications). Indeed, through the implementation of these solutions, almost all participants were able to navigate through the entirety of the study without any manual research staff intervention, and even if manual intervention was required, these applied experiences helped the research team develop a rigorous protocol of troubleshooting guidance for participants.

**Table 2:** Challenges encountered and solutions explored during the development and implementation of chatbot microintervention.

Challenge			Solutions explored
**Development**			
	Limits to how many consecutive messages or media files could be sent	Integrating more user-response questions Splitting up message clusters into smaller message groups Condensing and simplifying text messages	
	Limits to the formats and dimensions of media files were displayed on social media platforms	Choosing more square-like images as opposed to very wide/rectangular images Converting all images into PNG or JPEG format	
	Limits to the length and number of preset answer choices	Choosing “quick replies” (which allows for more answer choices) over “buttons” for user-response questions Condensing and simplifying preset answer choices (limiting to at most 6 answer choices) Integrating more open-response questions	
	Inability to provide preset answer choices on Desktop Instagram Messenger and Facebook Lite	Providing a notice in the chatbot welcome message on these specific incompatibilities and links for alternative versions of Facebook or Instagram to converse with the chatbot	
	Inability to return midway through a lesson after a problem was resolved	Creating checkpoints in the middle of lessons with keywords that, when texted, would allow participants to return to that checkpoint	
	Inability to manually launch a chatbot flow without going through the chatbot platform	Along with connecting flows to preset answer choices, each flow was set up to also initiate if a specific keyword was texted (eg, “get started” and “return”)	
**Implementation**			
	Chatbot stopped speaking due to server overburden	Splitting lessons into two separate, sequential chatbot flows Not forcing the chatbot to wait indefinitely for each user response without mechanisms to continue to the next message Reducing the size and resolution of media files Informing participants of the potential of chatbot disruptions, providing multiple self-administered troubleshooting strategies, and regularly monitoring conversations to provide manual guidance if needed	
	Chatbot stopped speaking due to sporadic social media or chatbot platform glitches	Checking updates from the chatbot company website and emails on temporary systemwide outages or glitches Joining a Facebook group of other users of the chatbot company to check on system outages or glitches and learn strategies to improve the chatbot experience Informing participants of the potential of chatbot disruptions, providing multiple self-administered troubleshooting strategies, and regularly monitoring conversations to provide manual guidance if needed	
	Chatbot stopped speaking due to participant not selecting a preset answer	Enforcing response validation for “quick reply” questions, which provided a reminder to choose from preset answer choices Informing participants of the potential of chatbot disruptions, providing multiple self-administered troubleshooting strategies, and regularly monitoring conversations to provide manual guidance if needed	
	Participant is unable to start a conversation with the chatbot using the link provided.	Providing backup instructions for manually locating the chatbot’s Facebook or Instagram page and initiating a chat Regularly monitoring conversations for chatbot initiation failure (eg, “get started” was not texted) and providing manual guidance if needed	
	Participant speaking with the chatbot from multiple accounts	Ensuring participant self-reported their social media handle in both the pretest and posttest surveys Messaging participant and using time-stamped message and profile information to confirm discrepancies	
	Participant accidentally starts a flow by entering a flow-initiating keyword	Ensuring flow-initiating keywords were both easy to remember and unlikely to be used otherwise during chatbot conversations (eg, “grapes1” instead of “grapes”)	

## Discussion

### Principal Findings

By systematically documenting the procedures taken to develop this social media chatbot microintervention along with analyzing both participant and researcher perspectives on its implementation, this study revealed that social media chatbots could indeed feasibly and effectively be used to provide rapid, tailored health education. Overall, participants as a collective reported a generally positive experience with the chatbot (particularly with respect to its pragmatic quality) and displayed moderately high engagement, although they provided some recommendations aimed at making the chatbot more interactive and personalized. Challenges were encountered during both the chatbot development and implementation, although the solutions developed were successful in maintaining implementation efficiency and a smooth experience for participants. By carefully describing these applied user and researcher experiences and outlining tested solutions to problems that were encountered, we provided a practical blueprint for public health professionals interested in leveraging social media chatbots as a medium for health education.

### Comparison With Previous Work

Findings related to the participant experience and feasibility of the social media chatbot support emerging research similarly suggesting that social media chatbots are effective, engaging ways of receiving health information [10], particularly among younger participants [45] (who, in this study, were observed to reported higher hedonic quality, ie, found the chatbot to be particularly fun and interesting). Furthermore, a notable finding was that participants felt the chatbot offered a powerful, pragmatic use, supported by the strong agreement that the chatbot helped participants learn new, helpful, and relevant information. However, to build on these preliminary findings, future comparative research is now warranted to examine both the pragmatic and hedonic qualities of similar rule-based chatbots, as well as their feasibility in comparison with both standard methods of health education (eg, in-person instruction) or more human-based chatbots (which may be less consistent and standard in the health education provided but may display higher hedonic quality).

Given that a recent systematic review of AI-based chatbots revealed that only 4 of the 15 examined studies reported technical difficulties experienced during chatbot participation [46], an important contribution of this study is the characterization of challenges and technical difficulties experienced during the study (both from the perspective of participants and research staff) and, importantly, the solutions developed to meet obstacles. Technical difficulties can have significant consequences in derailing both a chatbot’s feasibility and impact [46,47]; however, the solutions presented in this study reveal that various steps can be taken during the development and implementation of the study to provide multiple fail-safes against both foreseen and unforeseen obstacles. Specifically, a keen awareness of the dynamic policies, limitations, and complexities of Facebook, Instagram, and Manychat (the third-party chatbot software chosen for the study) was crucial to ensure the chatbot’s smooth implementation and will also be critical for the development of similar chatbot microinterventions using these platforms. Nonetheless, the challenges and solutions described in this study are certainly not exhaustive; future implementation of more scaled-up, complex chatbot interventions warrants similar exploration of other methods of minimizing implementation obstacles and enhancing user experience.

A notable suggestion made by some participants for future iterations of similar social media chatbot microinterventions is the greater integration of AI in the chatbot to enhance the personalization and tailoring of lesson content and engagement. Indeed, with the rise of advanced and easily accessible AI software such as ChatGPT, including as part of health research, there is a crucial need to consider the implications of such software as part of social media chatbot interventions [48]. Past research has explored AI-integrated chatbot tools to offer customized meal plans, personalized nutrition guidance, and ongoing monitoring of dietary habits [49]. These tools use data from diverse sources, such as health records and lifestyle surveys, to generate personalized nutrition plans and identify possible nutrient imbalances or deficiencies [49]. Indeed, AI has the unique potential to individualize health or diet education by considering an individual’s distinct needs, goals, and preferences. This personalized approach considers factors like lifestyle, medical history, cultural considerations, and personal tastes to provide customized recommendations [37]. For example, there were some differences among participants on what tenor of chatbot conversation style would resonate most with them (eg, some suggested using more emojis and some suggesting less); an AI-integrated chatbot may be able to leverage information on the participant’s background (age, gender, ethnicity, etc) to not only tailor the course content but perhaps even its conversation style. In addition, AI-powered tools can promote accountability and consistency by enabling users to track their habits, recognize patterns, and make informed adjustments to their dietary choices [10].

However, it is also important to consider the potential challenges of integrating AI into social media chatbots to provide the type of rapid educational microintervention explored in this study. For example, health education on topics such as diet requires a cautious, nuanced approach to ensure information is being provided in a way that both encourages healthy eating behaviors and does not unintentionally condone unhealthy relationships with food; indeed, for this reason, all chatbot messages were reviewed and revised by a registered dietician before study implementation. Conversely, AI-based responses may entail potentially problematic inconsistency in language compared to responses from text messages designed by individuals with formal health training [48]. Furthermore, personalization in responses also poses the risk of AI-based chatbots being unable to interpret certain user responses; Fadhil et al [50] pointed out such limitations in the interaction with chatbots, particularly regarding managing singular and plural conversational forms. Finally, another set of challenges for AI-integrated chatbots lies in ensuring that privacy and data security can be maintained while allowing AI systems to digest and interpret user response data [51]. Indeed, as AI technology continues to improve rapidly, it will be important to consider how these improvements can address these existing challenges with AI integration into social media chatbot interventions.

Furthermore, the ethics of how best to use social media as part of health research continues to be an emerging and complexifying discourse [52]; for example, unlike with researcher-collected survey data, researchers may not be able to maintain complete control of the privacy of social media data. As such, similar to how it was done with this study, researchers interested in implementing chatbots must carefully decide which types of data they will be collecting (including which platforms) and carefully communicate this information, for example, by making a note of both information that will be analyzed and information that will visible even if not analyzed. Researchers who decide to analyze social media data themselves must take extra steps to communicate data privacy limitations.

### Strengths and Limitations

Although the study’s strengths lie in its novel exploration of a standardized educational microintervention and comprehensive implementation evaluation from both participant and researcher perspectives, leveraging both quantitative and qualitative data, some limitations must be acknowledged. First, this study was conducted among a convenience sample of South Asian American young adults and centered around dietary behaviors; further research will benefit from implementing similar chatbots in other populations and on other health topics to corroborate usability and feasibility findings. Second, although steps were taken to enhance the reliability of the data collected, and social media– or engagement-related metadata were also used to assess user experiences and identify potential fraudulent responses, the study still relied upon self-reported data with a constrained number of quantitative and qualitative variables, and some participants could have evaded these data quality safeguard measures; future research may benefit from exploring a more expanded survey or conducting additional identity verifications (eg, assessing IP addresses and geolocation) to further minimize recruitment and data quality variability. Third, although research staff did make qualitative observations of responses to the chatbot to identify any participants providing clearly random or irrelevant responses to the chatbot, and such responses were not included in engagement calculations, systematic qualitative analyses of this content were not conducted to maintain the anonymity of participants (as the chatbot asked questions to personalize and contextualize lesson content) and to avoid analyzing social media message data itself (given potential privacy concerns); future research may benefit from exploring AI-integration or qualitative content analyses of responses to chatbots to better capture variability in chatbot engagement. Fourth, both social media and chatbot platforms often face dynamic changes in structure and policies, and thus, the functionalities that were explored in the chatbot developed for this study may differ from those offered in other types of chatbots, other platforms, or future versions of Facebook or Instagram.

### Future Directions

Overall, social media chatbots hold enormous potential to be leveraged by health educators to provide rapid, tailored, and impactful interventions in a manner that is neither time- nor resource-intensive. By systematically documenting the development and implementation of this chatbot, this study provides a blueprint for other researchers to build and evaluate similar chatbots. In addition, by highlighting both the practical use of the chatbot as well as integrating both researcher and participant perspectives to describe its usability and feasibility, this study provides the following recommendations for future research and practice: (1) testing scaled-up interventions to assess whether a similar rule-based health education chatbot shows strong user experience and engagement in other populations or on other health topics and (2) leveraging AI or other new chatbot features to improve user experience and engagement.

## Appendix

Multimedia Appendix 1 Characteristics of participants of the “Roti” social media chatbot intervention (n=168).

Multimedia Appendix 2 Differences in the positive and negative/neutral user experiences of the “Roti” social media chatbot microintervention (n=116).

## Abbreviations

AI: artificial intelligence
STC: Starting The Conversation
TPB: Theory of Planned Behavior
UEQ: User Experience Questionnaire
