# Supplementary material for: Rapid, Tailored Dietary and Health Education Through A Social Media Chatbot Microintervention: Development and Usability Study With Practical Recommendations
Source: JMIR Form Res. 2024 Dec 9;8:e52032. doi: 10.2196/52032 (PMC11667145; doi:10.2196/52032)
Supplement: Multimedia Appendix 1 [file formative_v8i1e52032_app1.docx]

| **Multimedia Appendix 1. Characteristics of participants of the “Roti” social media chatbot intervention (n=168)** | |
| --- | --- |
|  | Participants |
| Age, mean (SD) | 24.53 (3.14) |
| Sex, n (%) |  |
| Female | 129 (76.8) |
| Male | 39 (23.2) |
| Country of birth, n (%) |  |
| Outside of US | 95 (56.5) |
| US | 73 (43.5) |
| Education |  |
| Master’s or above | 89 (53.0) |
| Bachelor’s | 48 (28.6) |
| Some college / Associate’s | 18 (10.7) |
| High school | 13 (7.7) |
| South Asian ethnicity, n (%) |  |
| Indian | 119 (70.8) |
| Pakistani | 22 (13.1) |
| Bangladeshi | 24 (14.3) |
| Other | 6 (3.6) |
| Acculturation score, mean (SD) | 3.89 (0.65) |
| Diet health (STC) score, mean (SD) | 6.94 (2.14) |
| Platform used to participate |  |
| Facebook | 92 (54.8) |
| Instagram | 76 (45.2) |
| Total lessons, mean (SD) | 2.58 (1.25) |
| Total lesson time (min), mean (SD) | 34.39 (18.61) |
| Avg. time / lesson (min), mean (SD) | 13.87 (4.69) |
| Avg. engagement, mean (SD) | 0.75 (0.32) |
| ^a^ User Experience Questionnaire (UEQ), Range: -3 to 3 |  |
| Overall | 1.35 (1.31) |
| Pragmatic | 1.59 (1.37) |
| Hedonistic | 1.11 (1.42) |
| ^a^ Overall experience, Range: 1 to 10 |  |
| Learned something new | 8.24 (2.27) |
| Learned something helpful | 8.28 (2.31) |
| Relevant to me | 8.53 (2.08) |
| Would use it again | 7.27 (3.01) |
| Prefer to other ways of getting nutrition info | 7.30 (2.73) |
| Use to get info for other health topics | 7.72 (2.65) |

^a^ n=116
