# Supplementary material for: Rapid, Tailored Dietary and Health Education Through A Social Media Chatbot Microintervention: Development and Usability Study With Practical Recommendations
Source: JMIR Form Res. 2024 Dec 9;8:e52032. doi: 10.2196/52032 (PMC11667145; doi:10.2196/52032)
Supplement: Multimedia Appendix 2 [file formative_v8i1e52032_app2.docx]

**Multimedia Appendix 2. Differences in the positive and negative/neutral user experiences of the “Roti” social media chatbot micro-intervention (n=116)**

|  | **Overall UEQ** | | | **Pragmatic UEQ** | | | **Hedonistic UEQ** | | |
| --- | --- | --- | --- | --- | --- | --- | --- | --- | --- |
|  | Neut/Neg | Pos | p-value | Neut/Neg | Pos | p-value | Neut/Neg | Pos | p-value |
| Age, mean (SD) | 25.09 (3.02) | 24.20 (3.34) | 0.179 | 25.25 (2.90) | 24.22 (3.34) | 0.144 | **25.15 (3.03)** | **23.91 (3.36)** | **0.040** |
| Sex, n (%) |  |  |  |  |  |  |  |  |  |
| Female | 26 (74.3) | 67 (82.7) | 0.429 | 21 (75.0) | 72 (81.8) | 0.606 | 41 (78.8) | 52 (81.2) | 0.929 |
| Male | 9 (25.7) | 14 (17.3) |  | 7 (25.0) | 16 (18.2) |  | 11 (21.2) | 12 (18.8) |  |
| Country of birth, n (%) |  |  |  |  |  |  |  |  |  |
| Outside of US | 19 (54.3) | 44 (54.3) | 1.000 | 16 (57.1) | 47 (53.4) | 0.898 | 28 (53.8) | 35 (54.7) | 1.000 |
| US | 16 (45.7) | 37 (45.7) |  | 12 (42.9) | 41 (46.6) |  | 24 (46.2) | 29 (45.3) |  |
| Education |  |  |  |  |  |  |  |  |  |
| Master's or above | 20 (57.1) | 39 (48.1) | 0.794 | 17 (60.7) | 42 (47.7) | 0.429 | 31 (59.6) | 28 (43.8) | 0.113 |
| Bachelor's | 9 (25.7) | 23 (28.4) |  | 8 (28.6) | 24 (27.3) |  | 15 (28.8) | 17 (26.6) |  |
| Some college / Associate's | 4 (11.4) | 11 (13.6) |  | 2 ( 7.1) | 13 (14.8) |  | 4 ( 7.7) | 11 (17.2) |  |
| High school | 2 ( 5.7) | 8 ( 9.9) |  | 1 ( 3.6) | 9 (10.2) |  | 2 ( 3.8) | 8 (12.5) |  |
| South Asian ethnicity, n (%) |  |  |  |  |  |  |  |  |  |
| Indian | 23 (65.7) | 59 (72.8) | 0.581 | 19 (67.9) | 63 (71.6) | 0.889 | 37 (71.2) | 45 (70.3) | 1.000 |
| Pakistani | 7 (20.0) | 9 (11.1) | 0.327 | 6 (21.4) | 10 (11.4) | 0.303 | 9 (17.3) | 7 (10.9) | 0.472 |
| Bangladeshi | 5 (14.3) | 12 (14.8) | 1.000 | 3 (10.7) | 14 (15.9) | 0.711 | 6 (11.5) | 11 (17.2) | 0.554 |
| Other | 1 ( 2.9) | 2 ( 2.5) | 1.000 | 1 ( 3.6) | 2 ( 2.3) | 1.000 | 1 ( 1.9) | 2 ( 3.1) | 1.000 |
| Acculturation score, mean (SD) | 3.86 (0.62) | 3.93 (0.67) | 0.644 | 3.91 (0.66) | 3.91 (0.66) | 0.975 | 3.97 (0.64) | 3.86 (0.67) | 0.385 |
| Diet health (STC) score, mean (SD) | 6.40 (2.32) | 7.06 (2.01) | 0.124 | 6.32 (2.36) | 7.03 (2.03) | 0.122 | 6.71 (2.04) | 6.98 (2.19) | 0.493 |
| Platform used to participate |  |  |  |  |  |  |  |  |  |
| Facebook | 22 (62.9) | 38 (46.9) | 0.169 | 17 (60.7) | 43 (48.9) | 0.381 | 31 (59.6) | 29 (45.3) | 0.178 |
| Instagram | 13 (37.1) | 43 (53.1) |  | 11 (39.3) | 45 (51.1) |  | 21 (40.4) | 35 (54.7) |  |
| Total lessons, mean (SD) | 2.97 (1.10) | 3.21 (1.05) | 0.269 | 3.00 (1.12) | 3.18 (1.05) | 0.433 | 3.13 (1.01) | 3.14 (1.11) | 0.976 |
| Total lesson time (min), mean (SD) | 42.14 (20.62) | 40.15 (16.77) | 0.585 | 42.18 (20.44) | 40.30 (17.19) | 0.631 | 43.81 (19.48) | 38.27 (16.34) | 0.098 |
| Avg. time / lesson (min), mean (SD) | 14.33 (4.90) | 12.88 (4.25) | 0.110 | 14.29 (4.74) | 13.01 (4.39) | 0.189 | 14.11 (4.76) | 12.67 (4.18) | 0.086 |
| Avg. engagement, mean (SD) | 0.77 (0.30) | 0.85 (0.24) | 0.121 | 0.74 (0.33) | 0.85 (0.23) | 0.059 | 0.78 (0.28) | 0.86 (0.24) | 0.108 |
| Overall experience |  |  |  |  |  |  |  |  |  |
| Learned something new | **7.09 (2.85)** | **8.74 (1.76)** | **<0.001** | **7.11 (2.99)** | **8.60 (1.87)** | **0.002** | **7.21 (2.75)** | **9.08 (1.30)** | **<0.001** |
| Learned something helpful | **6.89 (2.97)** | **8.89 (1.64)** | **<0.001** | **6.96 (3.18)** | **8.70 (1.78)** | **<0.001** | **7.17 (2.73)** | **9.19 (1.37)** | **<0.001** |
| Relevant to me | **7.34 (2.79)** | **9.04 (1.44)** | **<0.001** | **7.43 (3.07)** | **8.88 (1.51)** | **0.001** | **7.56 (2.59)** | **9.31 (1.05)** | **<0.001** |
| Would use it again | **5.34 (3.11)** | **8.10 (2.58)** | **<0.001** | **5.29 (3.16)** | **7.90 (2.69)** | **<0.001** | **5.46 (3.04)** | **8.73 (2.06)** | **<0.001** |
| Prefer to other methods of nutrition info | **5.31 (2.69)** | **8.16 (2.27)** | **<0.001** | **5.68 (2.86)** | **7.82 (2.49)** | **<0.001** | **5.54 (2.69)** | **8.73 (1.76)** | **<0.001** |
| Use to get info for other health topics | **5.71 (2.82)** | **8.58 (2.05)** | **<0.001** | **5.75 (2.89)** | **8.34 (2.25)** | **<0.001** | **6.12 (2.90)** | **9.02 (1.49)** | **<0.001** |
